# Pathological and Immunological Developments in Behcet's Disease

**DOI:** 10.1155/2012/305780

**Published:** 2012-05-09

**Authors:** Umit Tursen, Gamze Piskin, Torello Lotti, Fereydoun Davatchi

**Affiliations:** ^1^Department of Dermatology, Mersin Medical School, University of Mersin, 33079, Mersin, Turkey; ^2^Department of Dermatology, Bovenij Ziekenhuis, Statenjachtstraat 1, 1034 CS Amsterdam, Postbus 37610, 1030 BD Amsterdam, The Netherlands; ^3^Department of Dermatology, University of Florence, Via della Pergola 60, 50132 Florence, Italy; ^4^Rheumatology Research Center, Shariati Hospital, Kargar Avenue, Tehran144114, Iran

Behcet's disease is a rare form of vasculitis that may have systemic multiorgan involvement. Behcet's disease was first defined by Hulusi Behcet, a Turkish Professor of Dermatology, in 1937 as a triad of recurrent aphthous stomatitis, genital aphthae, and relapsing uveitis. As this disease can be fatal, an immediate medical treatment is mandatory. So far there is no specific pathological testing or technique available for the diagnosis of the disease, although the International Study Group criteria for the disease are of good sensitivity and specificity. However, quite a portion of patients are misdiagnosed or have been delayed diagnosis. During the ensuing 65 years, multiple systemic associations of the disease including articular, vascular, gastrointestinal, cardiopulmonary, and neurologic involvement have become increasingly apparent. Although the etiology and pathogenesis is not clearly defined, genetic predisposition, infections, and immunological dysfunctions have been implicated. Behcet's disease has been reported worldwide but has a distinct geographic distribution, with highest prevalences in countries along with the ancient silk route. Although much has been learned during recent years on the pathogenesis and treatment of the disease, it is still an important cause of morbidity and mortality in areas where it is prevalent.

The entitled “*Musculoskeletal findings in Behcet's disease*” addresses the musculoskeletal findings of Behcet's disease, the relationship between Behcet's disease and spondyloarthropathy disease complex, and the status of bone metabolism in patients with Behcet's disease.

Professor Fereydoun Davatchi presents us the new diagnosis/classification criteria for Behcet's disease. The author finds that ICBD criteria have better sensitivity and accuracy than ISG. In the paper is entitled “*Pathophysiology of Behcet's disease*”, and we think that there is some clinical evidence suggesting that emotional stress and hormonal alterations can influence the course and disease activity of BD. Professor Erkan Alpsoy presents that the new evidence-based treatment is mainly based on the suppression of inflammatory attacks of the disease using immunomodulatory and immunosuppressive agents. In this paper, current state of knowledge regarding the therapeutic approaches is outlined. To provide a rational framework for selecting the appropriate therapy along with the various treatment choices, a stepwise, symptom-based, evidence-based algorithmic approach was developed. The fifth paper entitled “*Genetics of Behçet's disease*” describes HLA and non-HLA genetic association studies in BD. In recent years, several genomewide association studies and genetic polymorphism studies have also found new genetic associations with BD, which may have a supplementary role in disease susceptibility and/or severity. The paper entitled “*Histopathological evaluation of Behcet's disease and identification of new different skin lesions*” proposes that there has been an increase in the studies focusing on the histopathological aspects of Behcet's disease diagnostic mucocutaneous lesions. Their results emphasize the value of histopathology and direct immunofluorescence (DIF) in the differential diagnosis of Behcet's disease.

Tuba Kara and Duygu Dusmez Apa discuss the pathologic features of BD in the tubuler gut. Professor Aysin Kokturk describes that the differential diagnosis depends on a careful evaluation of the medical history and meticulous physical examination to detect concomitant systemic manifestations. Sometimes, some laboratory test may help establish the diagnosis. Subspecialty referral to ophthalmology, rheumatology, neurology, and gastroenterology should be considered when indicated. The paper about BD and endocrine system shows that the avaliable data suggest that there is an increased susceptibility to insulin resistance in patients with BD. And also the authors indicate that not only anterior hypophysis functions but also posterior hypophysis functions can be affected. As BD is a disease of autoimmune process, it may be possible that adrenal insufficiency or alterations in the cortisol levels could be expected.

The article entitled “*Potential infectious etiology of Behcet's disease*” indicates that an infectious agent could operate through molecular mimicry, and subsequently the disease could be perpetuated by an abnormal immune response to an autoantigen in the absence of ongoing infection. Potentia bacteria are *Saccharomyces cerevisiae*, mycobacteria, *Borrelia burgdorferi*, *Helicobacter pylori*, *Escherichia coli*, *Staphylococcus aureus*, and *Mycoplasma fermentans*, but the most commonly investigated microorganism is *Streptococcus sanguinis.* The relationship between streptococcal infections and Behcet's disease is suggested by clinical observations that an unhygienic oral condition is frequently noted in the oral cavity of Behcet's disease patients. Several viral agents, including herpes simplex virus-1, hepatitis C virus, parvovirus B19, cytomegalovirus, Epstein-Barr virus, and varicella zoster virus, may also have some role.

Professor Ozlem Yildirim shows that animal models allow for a more effective investigations into BD. The paper entitled “*Development of immunopathogenesis strategies to treat Behçet's disease*” finds that understanding of the new pathogenic mechanisms based on molecular structure of the disease helps us in improving the novel therapeutic modalities.

*Umit Tursen* *Umit Tursen* *Gamze Piskin* *Gamze Piskin* *Torello Lotti* *Torello Lotti* *Fereydoum Davatchi* *Fereydoum Davatchi*

